# Zglp-1 is a novel essential transcriptional regulator for sex reversal in zebrafish

**DOI:** 10.1007/s42995-025-00299-5

**Published:** 2025-05-12

**Authors:** Yajun Wang, Gaoqian Xu, Haoyi Li, Jing Gao, Xueqing Du, Wanyue Jiang, Guangdong Ji, Zhenhui Liu

**Affiliations:** 1 College of Marine Life Sciences, Key Laboratory of Evolution and Marine Biodiversity (Ministry of Education), Institute of Evolution and Marine Biodiversity, Ocean University of China, Qingdao, 266003 China; 2 Laoshan Laboratory, Qingdao, 266237 China

**Keywords:** Sex differentiation, Sex reversal, Zebrafish, ZGLP-1, SF-1, Zinc finger

## Abstract

Sex determination and differentiation play crucial biological roles in sexual reproduction in vertebrates, including zebrafish. Nevertheless, the intricate molecular mechanisms governing these processes have remained enigmatic. In this study, we showed a pivotal role played by zinc finger GATA-like protein-1 (Zglp-1) in sex differentiation in zebrafish. Our findings revealed that homozygous mutants having no Zglp-1 exhibited a female-to-male sex reversal, ultimately resulting in the development of fertile males. Within the pivotal phase of sexual differentiation, *zglp-1*^*−/−*^ zebrafish demonstrated a gene expression pattern that was skewed toward a male phenotype. Notably, the expression of *amh* was upregulated, while the expression of *cyp19a1a* was not sustained. Furthermore, our data revealed a direct interaction between the zinc fingers of Zglp-1 and Sf-1, which inhibited the ability of Sf-1 to activate the *amh* promoter. This interaction was crucial for regulating sex differentiation. Moreover, we observed alterations in gonadal cell proliferation and apoptosis in *zglp-1*^*−/−*^ zebrafish, which partially contributed to the sexual fate selection. Overall, our findings firmly established Zglp-1 as a crucial regulator of sex differentiation in zebrafish, offering deep insights into the intricate molecular mechanisms that govern sex determination and differentiation in vertebrates.

**Supplementary Information:**

The online version contains supplementary material available at 10.1007/s42995-025-00299-5.

## Introduction

Sex determination and differentiation are essential processes in animal reproduction and evolution. In mammals, the *SRY* gene, which is located on the Y chromosome, triggers the male developmental pathway within bipotential gonads (Bachtrog et al. 2014; Cutting et al. 2013; Gubbay et al. 1990; Koopman et al. 1991; Williams et al. 2009). Teleost fish, however, exhibit genetic sex determination with remarkable diversity, and is influenced both genetically and environmentally (Davidson et al. 2009; Devlin et al. 2002; Kitano et al. 2009; Ross et al. 2009; Ser et al. 2009; Siegfried et al. 2010; Volff et al. 2001). Zebrafish (*Danio rerio*), a representative teleost species, initially develop juvenile ovaries but eventually differentiate into two sexually distinct adult forms (Maack et al. 2003; Takahashi 1977; Uchida et al. 2002). While oocyte generation results in female maturation, males undergo apoptosis of their early-stage oocytes with subsequent testis development (Kossack et al. 2019). Furthermore, the maintenance of female fate depends on the presence of germ cells (Siegfried et al. 2008; Slanchev et al. 2005). It has been suggested that numerous genes are involved in the intricate processes of gonad development and sex determination (Cong et al. 2013; Hwang et al. 2013). However, the intricate gene networks governing these processes remain elusive.

Zinc finger GATA-like protein-1 (ZGLP-1), alternatively referred to as GLP-1, is a member of the GATA transcription factor family distinguished by a zinc finger motif at its C-terminus (Tevosian et al. 2002). Zglp-1 is predominantly expressed in Leydig cells of the testes and granulosa cells of the ovaries in mice (Li et al. 2007). Ablation of the *Zglp-1* gene leads to infertility in both male and female mice. In males, Zglp-1 deficiency leads to a significant reduction in mature spermatids, whereas in females it causes a complete loss of germ cells in embryonic ovaries (Li et al. 2007; Strauss et al. 2011). Zglp-1 has also been shown to induce primordial germ cell-like cells to differentiate into fetal oocytes by activating the ovulation program (Nagaoka et al. 2020). Although Zglp-1 is considered a conserved transcriptional regulator, its specific function in gonadal differentiation of zebrafish has not been investigated.

In our study, we created *zglp-1*^*−/−*^ zebrafish and discovered that they exclusively differentiated into males. The absence of females among these *zglp-1* mutants did not result from a lethal effect specific to the female sex, but rather from a female-to-male sex reversal phenomenon. This observation underscored the crucial role of Zglp-1 in promoting female differentiation in zebrafish. We also discovered that the zinc finger domain of zebrafish Zglp-1 interacted with the zinc finger domain of Sf-1, suppressing the transcription of anti-Müllerian hormone (*amh*). Hence, Zglp-1 is a newly discovered and critical transcriptional regulator that mediates sex reversal in zebrafish, offering valuable insight into the intricate mechanisms governing sex determination and differentiation in vertebrates.

## Materials and methods

### Cells

The HEK293T cell line, derived from human embryonic kidney cells, was obtained from American Type Culture Collection (ATCC, Manassas, Virginia, USA). The cells were cultivated in an incubator at 37 °C with 5% CO_2_ to ensure optimal growth. Cells were plated in Dulbecco’s modified Eagle medium (DMEM) supplemented with 10% fetal bovine serum (FBS) obtained from Gibco (Grand Island, NY, USA). To prevent bacterial contamination, antibiotics were incorporated into the medium at concentrations of 100 U/mL for penicillin and 100 μg/mL for streptomycin (MedChemExpress, Shanghai, China).

### Zebrafish

The zebrafish strains were maintained at a constant temperature of 28 °C, under a regimented photoperiod of 14 h of light alternating with 10 h of darkness. To create mutant lines, we used the CRISPR/Cas9 system targeting specific sequences for *zglp*-1 (5’-AGTGTGTGTGCCGCCGG-3’) and *sf-1* (5’-GGACACATGTGCAGAGAGTC-3’). sgRNA and Cas9 mRNA were produced using T7 RNA polymerase and simultaneously introduced into zebrafish embryos during the single-cell stage. Figures 2 and 7 show the CRISPR targeting sites and mutated sequences, respectively.

### Sequence analysis

Protein sequence alignments were performed using Clustal W in DNAMAN (Lynnon Biosoft, San Ramon, CA, USA). The secondary structures of ZGLP-1 proteins from humans, mice, and zebrafish were investigated using the SMART program (EMBL Heidelberg, Heidelberg, Germany, http://smart.embl-heidelberg.de/). For insights into protein three-dimensional conformations, structure predictions were made using the PHYRE2 (http://www.sbg.bio.ic.ac.uk/phyre2/html/page.cgi?id=index) server (Structural Bioinformatics Group, Imperial College, London, United Kingdom). Moreover, chromosomal locations of homologous genes in these species were retrieved from Sequence Viewer (NCBI, Bethesda, MD, USA, http://www.ncbi.nlm.nih.gov/projects/sviewer) and Ensembl Genome Browser (Ensembl, Hinxton, Cambridge, United Kingdom, http://www.ensembl.org).

### Sex identification of zebrafish

For the identification of zebrafish sex, we relied on dimorphic morphological features, including body shape, fin color, and the presence of genital papillae. This assessment generally required observation under a stereomicroscope (Nikon, Tokyo, Japan) to ensure accurate visualization of these key features. In certain instances, histological examination was deemed essential for confirming the sex.

## Histological analysis combined with In Situ Hybridization (ISH) techniques

To evaluate the phenotypic sex of wild-type zebrafish and heterozygous and homozygous mutants at 6 mpf, we observed sexually dimorphic traits, such as anal fin color, genital papilla, and body shape. For a deeper understanding of sexual development and genotype differences, gonads were dissected from euthanized animals. The samples were immobilized in 4% PFA for a duration of 12 h, embedded in paraffin, and sliced into sections measuring 8 μm in thickness. Subsequently, the sections were prepared, dyed, and subjected to ISH (Rodriguez-Mari et al. 2005) for additional examination.

## RNA quantification

RNA was extracted from samples using an E.Z.N.A. Total RNA kit (Omega, #R6834-01, Norcross, GA, USA), followed by DNase I treatment to remove genomic DNA. cDNA was synthesized using a HiScript II kit (Vazyme, #R211-01, Nanjing, China). Gene expression was quantified by real-time PCR with ChamQ SYBR mix (Vazyme, #Q431-02, Nanjing, China) on an ABI 7500 system. Zebrafish actb1 served as the internal control. Relative changes in expression were determined using the 2^−ΔΔCt^ approach. All experimental procedures were conducted in triplicate and replicated three times. The sequences of the primers utilized are shown in Supplementary Table S1.

### Transcriptomics

Heterozygous mutant zebrafish were crossed to generate offspring raised to 19 days post-fertilization (dpf) for pre-sex differentiation and 35 dpf for post-sex differentiation. Genomic DNA was obtained from the caudal fin to identify wild-type and homozygous mutant individuals. Gonadal RNA, isolated using the TRIzol reagent, was subjected to transcriptome sequencing on the Illumina NovaSeq 6000 platform (Gene Company Limited, Hong Kong, China). For each genotype there were three biological replicates. Using the DEGseq R package, differential gene expression analysis was conducted to identify genes that were notably upregulated or downregulated in homozygous mutants when compared to wild-type zebrafish. The entire analysis was performed using BMKCloud (www.biocloud.net, Biomarker Technologies, Beijing, China).

### Plasmid construction

We conducted cloning experiments to investigate the functions of zebrafish *zglp-1* and *sf-1* and their various domains. ORFs of *zglp-1* and *sf-1* were cloned into a pcDNA3.1 vector. An *amh* promoter was inserted into the pGL3-basic vector to facilitate luciferase reporter assays. Zglp-1-specific domains, including the zinc finger (ZnF, aa 227–374) and N-terminal regions (N, aa 1–226), were cloned into a pcDNA3.1 for transcriptional activity assays. Zglp-1, Zglp-1-ZnF, and Zglp-1-N ORFs were also cloned into pCMV-C-HA vectors. Sf-1, its ZnF (aa 1–163), and C-terminal (C, aa 164–502) ORFs were cloned into pCMV-C-Myc vectors. Finally, *zglp-1* and *sf-1* ORFs were cloned into pcDNA3.1-eGFP and pcDNA3.1-mCherry vectors, respectively. All cloning was performed using the Mut Express II kit. Primer sequences are provided in Supplementary Table S1.

### Subcellular co-location

To investigate the subcellular localization and interaction of Zglp-1 and Sf-1, we simultaneously introduced Zglp-1-eGFP and Sf-1-mCherry plasmids into HEK-293 T cells using Lipofectamine 2000. After a 36-h incubation, the cells were immobilized, cleansed, and labeled with DAPI for nuclear identification. Confocal microscopy was used to capture images, revealing the subcellular positioning of Zglp-1 and Sf-1, as well as their possible interactions.

### Luciferase activity assays

HEK-293 T cells were seeded and incubated in 24-well plates for a duration of one night. Then, the cells were co-transfected with plasmids using Lipofectamine 2000 (Invitrogen, #11668019, Thermo Fisher Scientific, Waltham, MA, USA). Transcriptional activity was evaluated using a luciferase reporter assay. The luciferase activity of each plasmid was measured and normalized (Yeasen, #11402ES60, Yeasen Biotechnology, Shanghai, China). Primers for cloning the promoter are shown in Supplementary Table S1.

### Immunoblot analysis and co-immunoprecipitation

For the co-immunoprecipitation (Co-IP) assay, whole-cell extracts were collected 48 h following transfection and disrupted in an NP-40 buffer. After completion of centrifugation, the soluble protein fraction was incubated with protein A/G beads conjugated to specific antibodies (Santa Cruz, #sc-2003, Santa Cruz Biotechnology, Santa Cruz, CA, USA) overnight. The beads were then washed, and the bound proteins were released using an SDS sample buffer. Western blot analysis was conducted on both the immunoprecipitated proteins and the whole-cell lysates, employing specific antibodies and utilizing enhanced chemiluminescence for detection. The antibodies used in this study were supplied by Beyotime (HA tag, 1:1000, #AH158; Myc tag, 1:1000, #AM926, Beyotime Biotechnology, Shanghai, China) and Abcam (VeriBlot for IP detection, 1:5000, #ab131366, Thermo Fisher Scientific, Waltham, MA, USA).

### Statistical analysis

To ensure reproducibility, the assays were replicated three times technically and biologically. Statistical significance was evaluated using Prism 5, employing either one-way ANOVA or a two-tailed Student's t-test. Data are represented as mean ± SD. The significance thresholds are denoted as follows: *P < 0.05, **P < 0.01, and ***P < 0.001; ns indicates non-significance.

## Results

### Zglp-1 zinc finger domains exhibit strong conservation across vertebrates

**Fig. 1:**
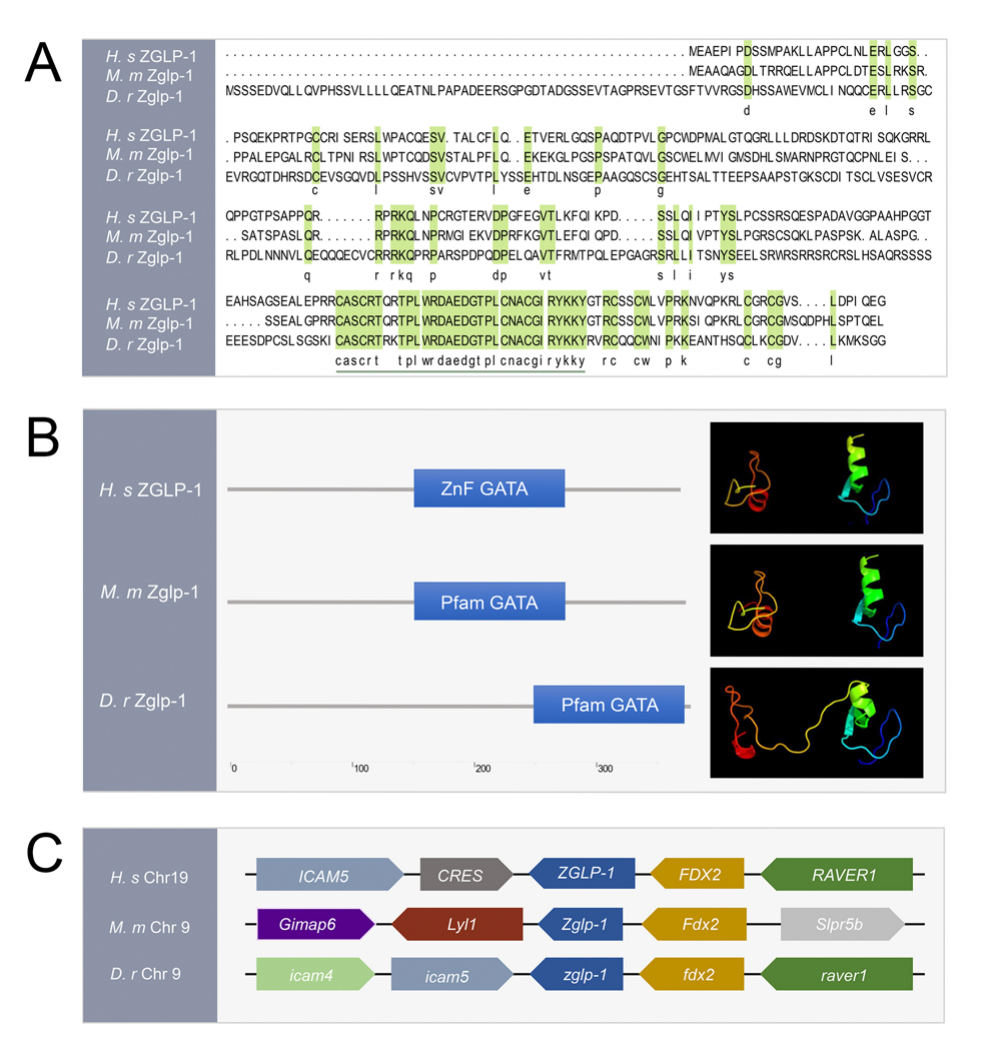
Zglp-1 zinc finger domains are well conserved in vertebrates. **A** Sequence alignment of the ZGLP-1 proteins. Amino acids that are identical are denoted by black letters, whereas those with a higher degree of conservation are represented by green background. Additionally, the sequences corresponding to the zinc finger domain are underlined for emphasis. **B** Comparison of ZGLP-1 proteins. The secondary structural details of ZGLP-1 proteins determined by employing the SMART program. Utilizing PHYRE2 analysis, the three-dimensional configurations of ZGLP-1 proteins from humans, mice, and zebrafish were predicted. **C** A syntenic map was crafted to depict the specific genomic region where zglp-1 is located within the chromosomes of zebrafish and other vertebrates. Rectangles denote genes, and the orientation of each rectangle signifies the transcriptional direction of the gene. Sequence source: *Homo sapiens* ZGLP-1*:* NP_001096637.1; *Mus musculus* Zglp-1: NP_001096638; *Danio rerio* Zglp-1*:* NP_001038914.1

The zebrafish *zglp-1* gene sequence (NCBI gene ID: 751739) was obtained from the National Center for Biotechnology Information (NCBI), accessible at https://www.ncbi.nlm.nih.gov. This gene encodes an mRNA transcript of 1373 base pairs translating into a protein comprising 374 amino acids. Sequence alignment using the Clustal W method within DNAMAN (Lynnon Biosoft, San Ramon, CA, USA) showed a high degree of conservation in the zinc finger domains of ZGLP-1 proteins across diverse species (Fig. 1A). The SMART program was used to conduct an analysis of the secondary structures of human, mouse, and zebrafish ZGLP-1 proteins, revealing the existence of a conserved GATA zinc finger domain across all three species (Fig. 1B). Moreover, the proteins from these species exhibited a striking similarity in their three-dimensional structures (Fig. 1B), highlighting the functional importance of this conserved domain. Syntenic analysis localized the zebrafish *zglp-1* gene to chromosome 6, where it was closely linked to the *fdx2* gene. The genomic organization observed in the zebrafish *zglp-1* gene reflected a similar pattern to that seen in human and mouse *ZGLP1* genes (Fig. 1C), suggesting that the linkage between *zglp-1* and *fdx2* has been maintained throughout vertebrate evolution. As a whole, the combined evidence from the data strongly indicated that the *zglp-1* genes in zebrafish and other vertebrate species are functionally conserved orthologs.

### The expression of zglp-1 is primarily observed in the ovary of zebrafish

Real-time PCR was used to investigate the tissue-specific expression of *zglp-1* in zebrafish. In particular, *zglp-1* showed its highest expression in the adult ovary (Supplementary Fig. S1A), while it was also detectable at lower levels in other tissues. This indicated a possible involvement of *zglp-1* in ovarian function. To further understand the temporal expression pattern of *zglp-1*, we analyzed its expression levels at different time points after fertilization (0, 5, 12, 18, 30, and 45dpf). Notably, *zglp-1* expression exhibited a significant increase at 30 dpf, a critical stage of gonad differentiation in zebrafish (Supplementary Fig. S1B). This expression pattern was strikingly similar to that of *amh*, a well-known sex determinant gene, further emphasizing the potential involvement of *zglp-1* in sex determination and gonadal development.

To localize the expression of *zglp-1* within the gonads, we employed paraffin sections for ISH. A pronounced positive signal for *zglp-1* was observed specifically in the oocytes of the ovary (Supplementary Fig. S1C), indicating a direct role in ovarian function. In contrast, a significantly fainter signal was observed in the spermatogonia of the testis, implying a reduced function or distinct regulatory mechanism in testicular tissue. Together, these findings implicated *zglp-1* as a key player in ovarian development and function in zebrafish.

### Knockout of zglp-1 leads to a full reversal of the sexual phenotype from female to male in zebrafish

**Fig. 2:**
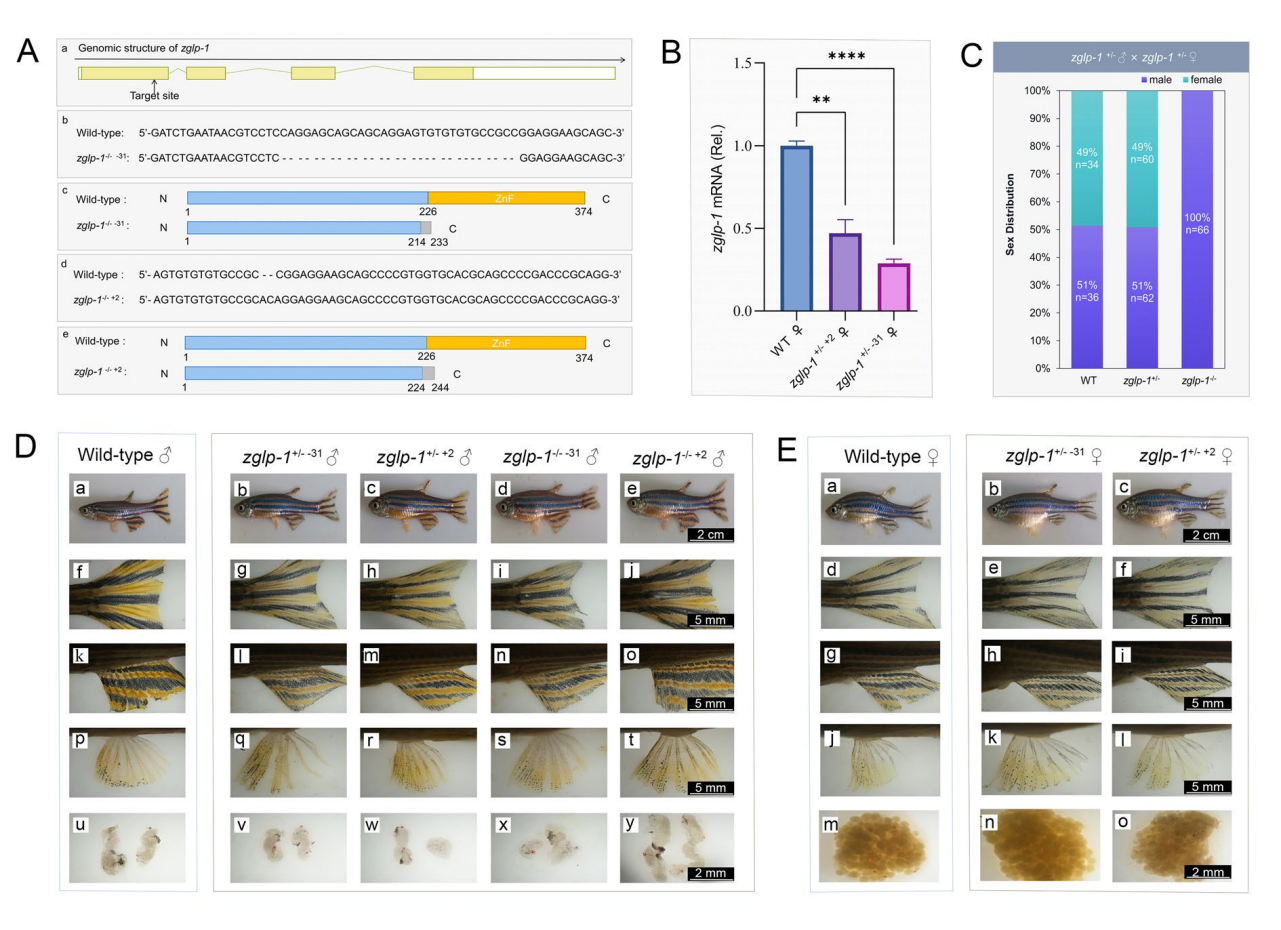
The lack of females observed in *zglp-1*^*−/−*^ mutants is attributed to sex reversal. **A** The CRISPR/Cas9 target location and zebrafish *zglp-1* mutant varieties. a. The intended knockout site for the *zglp-1* gene was located within exon 1, denoted by the boxed exons. The translated regions are marked by yellow squares, whereas the untranslated regions are indicated by white squares. The folded line represents introns. b and c. In comparison to the wild type, mutant 1 exhibited a deletion of 31 bases, resulting in premature translation termination at the 233rd amino acid. d and e. Mutant 2 differed from the wild type by the insertion of two bases and the substitution of one base, causing translation to halt prematurely at the 244th amino acid. **B** The expression of *zglp-1* in wild-type and *zglp-1* mutant zebrafish by quantitative real-time PCR. *actb1* served as the loading control in our study. The data presented here represent the mean values with standard deviations (mean ± SD). Statistical analysis was performed using one-way ANOVA. (**P < 0.01, ***P < 0.001). **C** Details of the counts (*n*) and proportions (%) of female and male offspring resulting from a cross between zglp-1 heterozygous mutant *zglp-1*^± +2^ males and *zglp-1*^± +2^ females, categorized accordingly. **D** Primary and secondary sex characteristics of *zglp-1* male mutants. The body (a–e), caudal fin (f–j), genital papilla (k–o), anal fin (p–t), and gonad at the cloaca (u–y) were observed (*n* = 10). **E** Primary and secondary sex characteristics of *zglp-1* female mutants. The body (a–c), caudal fin (d–f), genital papilla (g–i), anal fin (j–l), and gonad at the cloaca (m–o) were observed (*n* = 10)

To elucidate the functional significance of *zglp-1* in vivo, we generated *zglp-1*-deficient zebrafish using CRISPR/Cas9 technology. This approach allowed us to create two homozygous mutants, designated as *zglp-1*^−/− −31^ (mutant 1) and *zglp-1*^−/− +2^ (mutant 2). Both mutants encoded truncated products lacking the complete zinc finger domain (Fig. 2A), a key structural motif for protein-DNA interactions. Real-time PCR analysis confirmed a significant reduction in *zglp-1* expression in the ovaries of both mutant lines compared to that of the control (Fig. 2B), validating their defective status. To assess the phenotypic effects of *zglp-1* knockout, we bred *zglp-1*^±^ males and females from distinct mutant lineages. The offspring were reared until adulthood, and their *zglp-1* genotypes were identified through PCR analysis. For mutant 1, owing to the limited number of offspring obtained, phenotypic analysis was restricted. For mutant 2, we noted a genotype distribution among 258 offspring that aligned closely with the anticipated Mendelian ratio of 1:2:1 for wild-type zebrafish and heterozygous and homozygous mutants, respectively. This finding indicated that the *zglp-1* gene knockout did not result in lethality.

Next, we examined the sex ratio among wild-type, *zglp-1*^± +2^ heterozygous mutant, and *zglp-1*^−/− +2^ homozygous mutant zebrafish. Phenotypic sex was discerned by observing the coloration of the anal fin, the presence of a genital papilla, and overall body shape. Interestingly, although the male-to-female sex ratio in wild-type and heterozygous mutant zebrafish was approximately 1:1, all 66 homozygous mutant zebrafish were male (Fig. 2C). This finding was consistent across both mutant lines, with all *zglp-1*^−/− −31^ zebrafish also revealing male phenotypes. To ensure the accurate identification of sex characteristics, we performed dissections to confirm the primary sex characteristics of the zebrafish used in our study. All fish showed excellent concordance between their primary and secondary sex characteristics (Fig. 2D, E), ruling out any mismatch that could potentially confound our results. Moreover, we demonstrated that the sperm from male *zglp-1* homozygous mutant zebrafish was fertile, indicating that the sex reversal observed was physiologically functional. Our findings strongly suggest that knockout of *zglp-1* resulted in a full female-to-male sex reversal in zebrafish. This observation highlights the pivotal importance of *zglp-1* in ovarian development and the preservation of female sexual characteristics in this species.

### Knockout zglp-1 does not influence the expression of amh or cyp19a1a in 19-dpf zebrafish

At 19 dpf, the gonads of zebrafish have not yet undergone discernible morphological differentiation. To understand if the *zglp-1* gene plays a regulatory role in molecules linked to gonad differentiation during this developmental phase, transcriptome sequencing was conducted on total gonad RNA extracted from both wild-type and zglp-1 knockout (*zglp-1*^−/− +2^) zebrafish. Examination of the sequencing data revealed that 221 genes were upregulated and 355 genes were downregulated in response to the knockout of the *zglp-1* gene. These differentially expressed genes were primarily associated with reproductive and developmental signaling pathways, including MAPK, GnRH, ECM–receptor interactions, and arachidonic acid metabolism (Supplementary Fig. S2A). This finding indicates that, despite the absence of overt morphological changes in the gonads at this stage, *zglp-1* did have an impact on genes involved in cell growth and differentiation processes. Surprisingly, the expression levels of *amh* and *cyp19a1a*, two well-established sex-specific markers for somatic gonadal cells in the testis and ovary, respectively (Rodríguez-Marí et al. 2005; Siegfried et al. 2008; Yao et al. 2003), were not significantly altered in the *zglp-1* knockout zebrafish (Supplementary Fig. S2B, C). This suggests that *zglp-1* may regulate gonad differentiation via alternative pathways or may have a more subtle effect on these markers that was not detectable at this developmental time point.

### Lack of zglp-1 shows a male expression profile in 35-dpf zebrafish

**Fig. 3:**
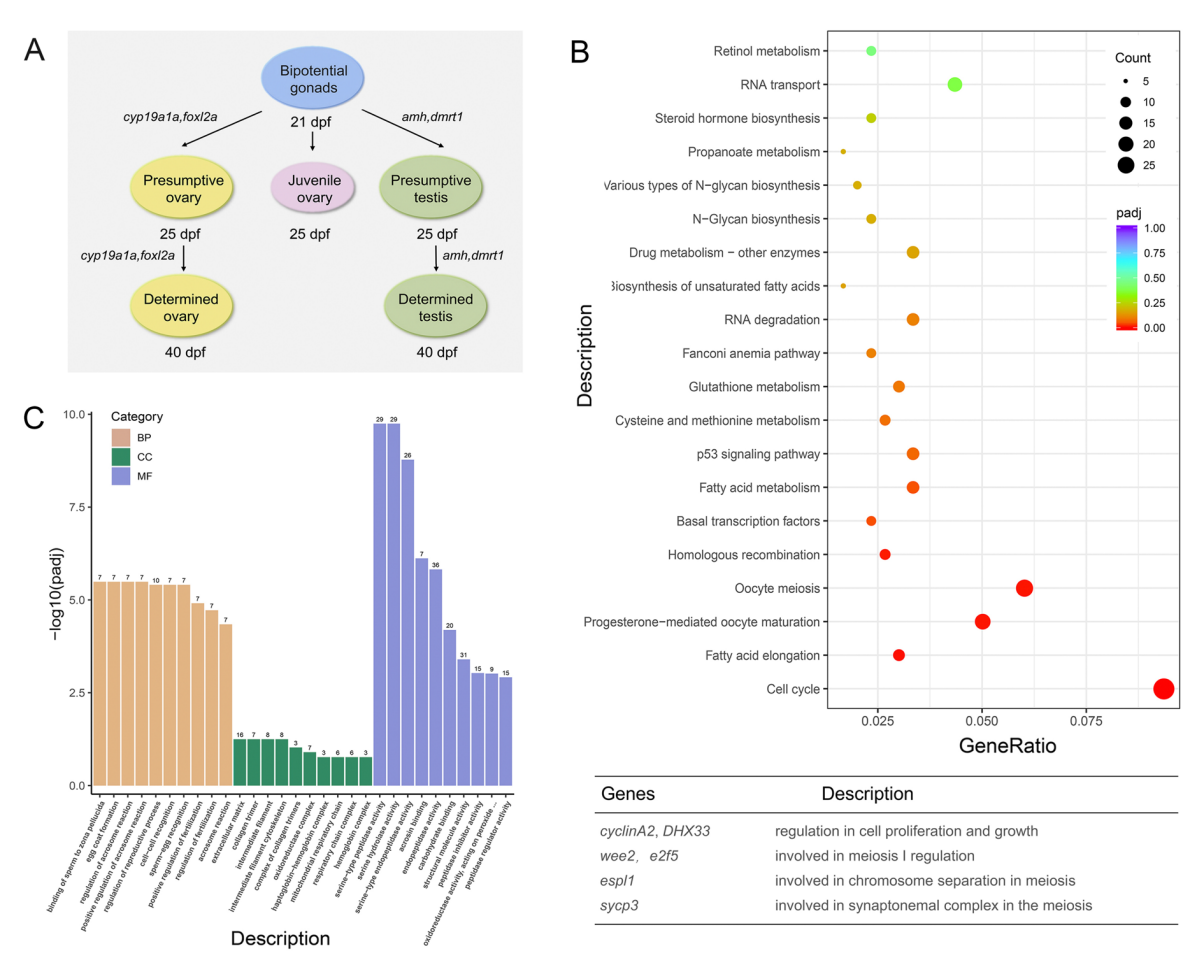
*zglp-1*^−/−^ mutants show a male expression profile. **A** Sex determination and gonad development in zebrafish. The development of gonads first passes through an ovary-like stage (Ye et al. 2020). **B** KEGG analysis of differentially expressed genes between wild-type female zebrafish and *zglp-1*^−/− +2^ zebrafish at 35 dpf. **C** GO analysis histogram of differentially expressed genes between wild-type male zebrafish and *zglp-1*^−/− +2^ zebrafish at 35 dpf

As described previously, all *zglp-1*^−/−^ mutants developed as males, leading us to hypothesize that knockout of *zglp-1* initiates the male pathway from the onset of gonad differentiation. To test this, we conducted a transcriptome sequencing analysis at 35 dpf, a stage by which wild-type juvenile zebrafish have passed the critical period of sex determination (Fig. 3A). At this stage of development, the distinction between males and females can be based on their respective expression levels of the male marker gene *amh* and the female marker gene *cyp19a1a*. We sequenced the transcriptomes of wild-type female, wild-type male, and *zglp-1*^−/− +2^ zebrafish and performed a comparative analysis.

We first investigated the genes exhibiting differential expression patterns between wild-type female zebrafish and those homozygous for the *zglp-1* mutation. It is noteworthy that the genes downregulated in the *zglp-1* mutants were significantly enriched in processes associated with the cell cycle, progesterone-mediated oocyte maturation, and oocyte meiosis (Fig. 3B). Key genes in this group included cyclin A2 and DHX33, integral to cell proliferation and growth, as well as wee2 and e2f5, modulators of meiosis I. Furthermore, espl1, which is essential for chromosome separation during meiosis, and sycp3, a critical component of the synaptonemal complex, were both downregulated. These findings indicate the likelihood that the knockout of *zglp-1* disrupted the development of the female reproductive pathway, potentially causing sexual reversal from female to male.

In addition, we conducted a comparison of differentially expressed genes between wild-type male zebrafish and *zglp-1* homozygous mutants. The downregulated genes in the mutants were predominantly associated with sperm binding to the zona pellucida and regulation of the acrosome reaction (Fig. 3C). This suggests subtle negative impacts on testicular structure and function, despite the fertility of *zglp-1*^−/−^ zebrafish. This finding was further supported by histological examination of the testes from these mutant adult zebrafish.

**Fig. 4:**
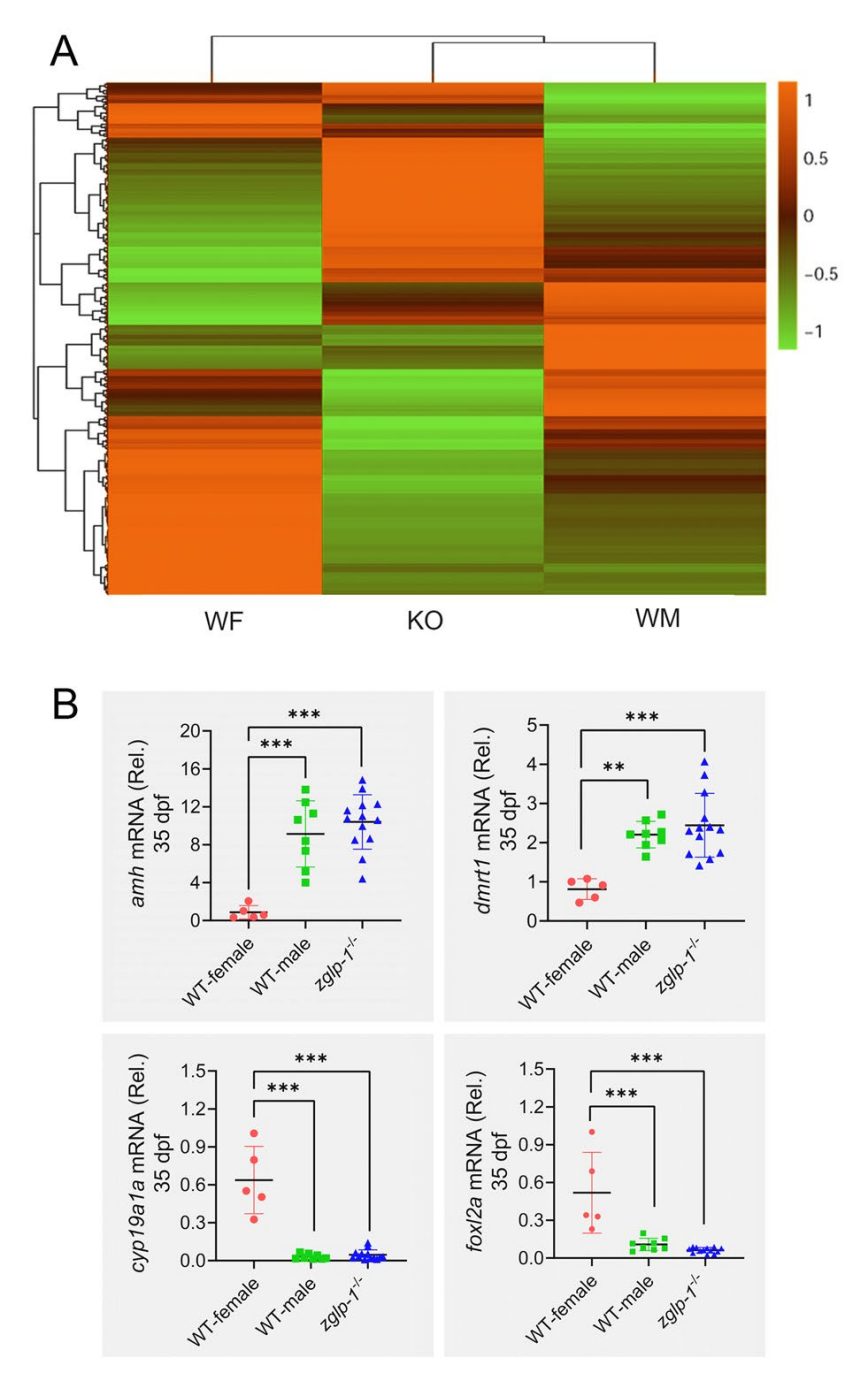
*zglp-1*^−/−^ mutants show a male expression profile. **A** A cluster analysis was conducted to identify differentially expressed genes among the three distinct groups: *zglp-1*^*−/−*^ zebrafish (KO), wild-type male zebrafish (WM), and wild-type female zebrafish (WF). **B** A comparative analysis was performed to assess the expression levels of *amh*, *dmrt1*, *cyp19a1a*, and *foxl2a* genes in WM, WF, and *zglp-1*^−**/−**^ zebrafish at 35 dpf. *actb1* was used as a control. Data are presented as mean ± SD. The data were subjected to statistical analysis using one-way ANOVA (**P < 0.01, ***P < 0.001)

Finally, a cluster analysis of the differentially expressed genes among the three groups revealed a stronger affinity between the *zglp-1*^−/−^ zebrafish and wild-type males than wild-type females (Fig. 4A). In accordance with this observation, all 35-dpf *zglp*-1^−/−^ mutants exhibited a distinct male-specific expression pattern, featuring upregulated expression of *amh* and *dmrt1*, and downregulated expression of *cyp19a1a* and *foxl2a* (Fig. 4B). Overall, our findings strongly implicated Zglp-1 as a critical regulator of sexual differentiation and gonad development in zebrafish.

### The ability of Sf-1 to activate the amh promoter is suppressed by the zinc finger domain of Zglp-1

**Fig. 5:**
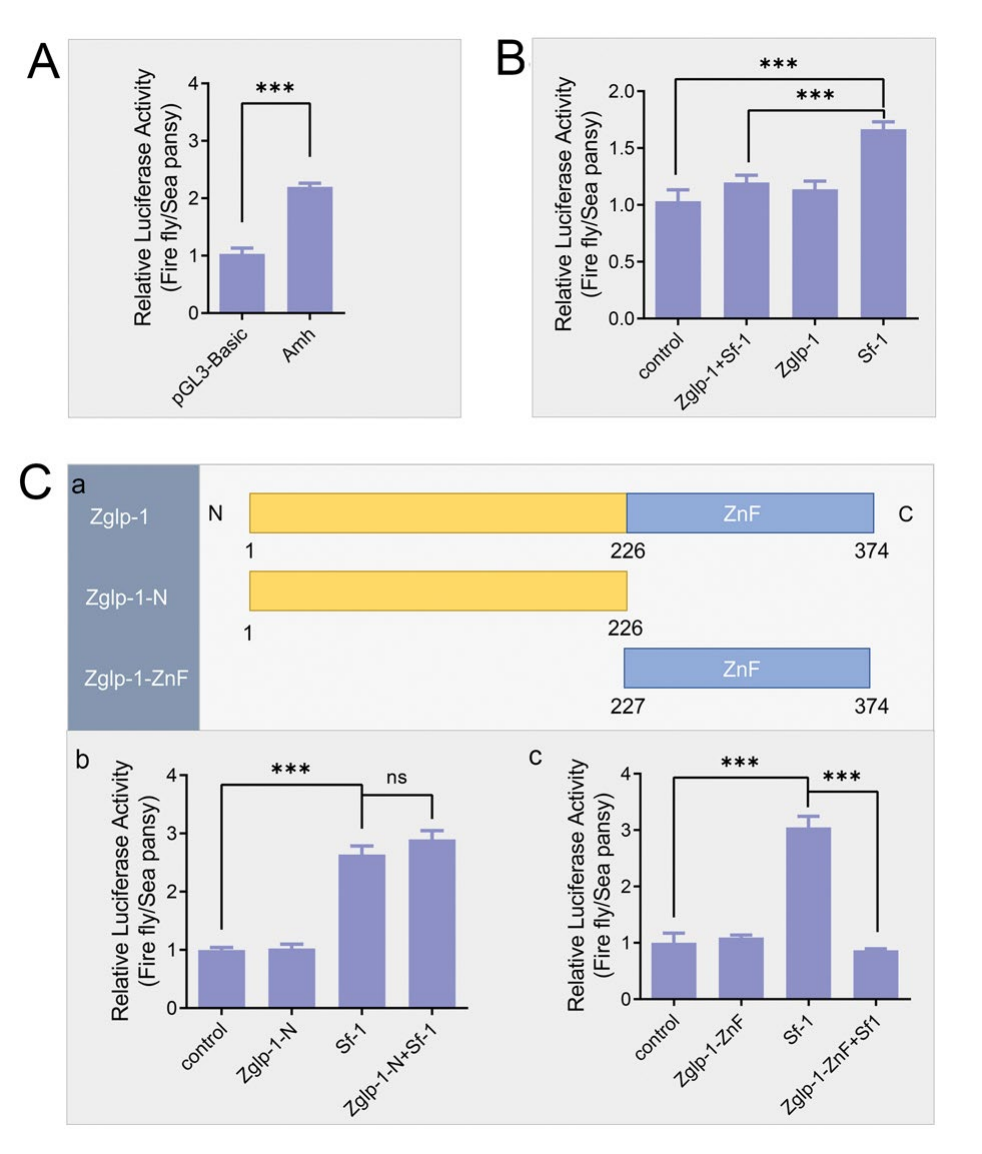
Zglp-1 inhibits the transcription of *amh* activated by Sf-1 in its zinc finger domain region. **A** *amh* promoter activity test. The *amh* promoter demonstrated transcriptional activity in contrast to the control. **B** The regulatory relationships among Zglp-1, Sf-1, and the *amh* promoter determined by a dual-luciferase reporter gene assay. The activation of the *amh* promoter was suppressed when Zglp-1 and Sf-1 were co-transfected into HEK293T cells. **C** The functional domain of Zglp-1 that blocks the ability of Sf-1 to activate the *amh* promoter. a. The domain structure of zebrafish Zglp-1, as predicted by the SMART program, revealed two distinct sections within its amino acid sequence. In particular, amino acids 227–374 constituted the Zglp-1-ZnF region, which encompassed the zinc finger domain. Amino acids 1–226 formed the Zglp-1-N segment. b. The regulatory relationships among Zglp-1-N, Sf-1, and the *amh* promoter. c. The regulatory relationships among Zglp-1-ZnF, Sf-1, and the *amh* promoter. Zglp-1-ZnF, rather than Zglp-1-N, suppressed the ability of Sf-1 to activate the *amh* promoter. Data are presented as the mean ± SD. ***P < 0.001; ns, no statistical difference

Considering the elevated *amh* expression observed in *zglp-1*^−**/−**^ zebrafish, we constructed an *amh* promoter with transcriptional activity (Fig. 5A). However, our dual-luciferase reporter gene assay failed to demonstrate that Zglp-1 directly regulated *amh* transcription (Fig. 5B). Previous studies have established steroid synthesis factor 1 (Sf-1) as a crucial regulator of gonad development, capable of activating *amh* transcription, a finding corroborated by our results (Fig. 5B). Intriguingly, when Zglp-1 and Sf-1 were co-transfected into HEK293T cells, the transcriptional activation of *amh* by Sf-1 was specifically repressed (Fig. 5B). Consequently, Zglp-1 appeared to impede Sf-1's ability to activate the *amh* promoter.

For a more comprehensive understanding of the functional domains of Zglp-1, we cloned both the zinc finger domain (Zglp-1-ZnF) and the amino-terminal region (Zglp-1-N), as illustrated in Fig. 5C-a. Luciferase assays showed that the zinc finger domain (Zglp-1-ZnF) significantly impaired Sf-1’s capacity to activate the *amh* promoter, whereas the Zglp-1-N region did not (Fig. 5C-b, c). Our results indicated that the zinc finger domain of Zglp-1 held a critical function in regulating *amh* transcription, which was facilitated by Sf-1.

### Zglp-1 interacts with Sf-1 in the nucleus, primarily through their zinc finger domains

**Fig. 6:**
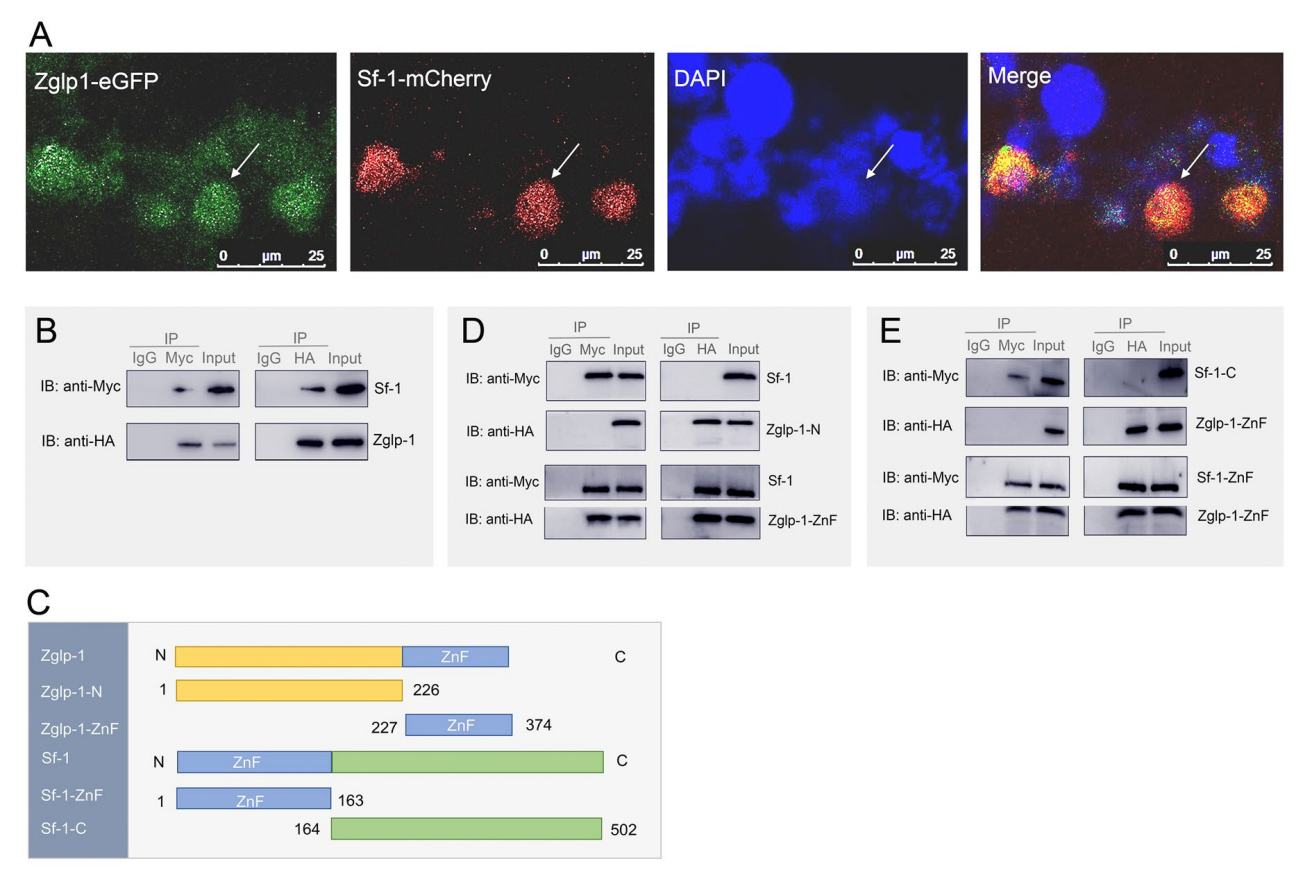
Interaction of Zglp-1 and Sf-1 occurs between their zinc finger domains. **A** Co-localization of Zglp-1 and Sf-1 was observed in the nucleus of HEK293T cells. Confocal microscopy images taken 24 h after co-transfection revealed the expression of Zglp-1 (depicted in green) and Sf-1 (shown in red) within the cells. Nuclei were counterstained with DAPI (blue). **B** An interaction analysis between Zglp-1 and Sf-1 was performed through co-immunoprecipitation and immunoblot assays in HEK293T cells co-transfected with Zglp-1-HA (2 μg) and Sf-1-Myc (2 μg). **C** Domain structure of zebrafish Zglp-1 and Sf-1 predicted by the SMART program. The amino acid sequence of Zglp-1 was divided into two sections. Amino acids 227–374 were designated Zglp-1-ZnF. Amino acids 1–226 were designated Zglp-1-N. The Sf-1 amino acid sequence was segmented into two distinct regions: amino acids 1–163, designated as Sf-1-ZnF, and amino acids 164–502, constituting the Sf-1-C segment. **D** Interaction studies were conducted in HEK293T cells co-transfected with combinations of Zglp-1-ZnF-HA (2 μg) and Sf-1-Myc (2 μg), or Zglp-1-N-HA (2 μg) and Sf-1-Myc (2 μg). These interactions were analyzed using co-immunoprecipitation and immunoblot techniques. **E** Similar interaction analyses were performed in HEK293T cells co-transfected with Sf-1-ZnF-Myc (2 μg) and Zglp-1-ZnF-HA (2 μg), or Sf-1-C-Myc (2 μg) and Zglp-1-ZnF-HA (2 μg). Co-immunoprecipitation and immunoblotting were employed to assess the interactions between these proteins

Previous data demonstrated that Zglp-1 suppressed Sf-1's ability to activate the *amh* promoter. This led us to investigate whether Zglp-1 and Sf-1 interacted within cells, particularly in the same subcellular region. Initially, we examined their expression and subcellular localization in HEK293T cells. As shown in Fig. 6A, our findings revealed the co-localization of Zglp-1 and Sf-1 proteins within the nucleus, hinting at a possible interaction between them. To confirm this interaction, we conducted co-IP experiments. The results presented in Fig. 6B confirmed that Zglp-1 interacted with Sf-1 in cells.

To further dissect the regions responsible for this interaction, we cloned two domains of Zglp-1: the zinc finger domain (Zglp-1-ZnF) and the amino-terminal region (Zglp-1-N) (Fig. 6C). Co-IP assays revealed that Zglp-1-ZnF, but not Zglp-1-N, directly interacted with Sf-1 (Fig. 6D). Similarly, we cloned the zinc finger domain (Sf-1-ZnF) and the carboxyl-terminal region (Sf-1-C) of Sf-1 (Fig. 6C). Our findings indicated that the interaction between Zglp-1 and Sf-1 primarily occurred through their zinc finger domains (Fig. 6E). Given that the zinc finger domain of Zglp-1 inhibited Sf-1's ability to activate the *amh* promoter, as shown previously, we reasonably inferred that the interaction between the zinc finger domains of Zglp-1 and Sf-1 was the key factor that hindered Sf-1's ability to transcriptionally activate the *amh* promoter.

### Sf-1 is crucial for gonad development in zebrafish

**Fig. 7:**
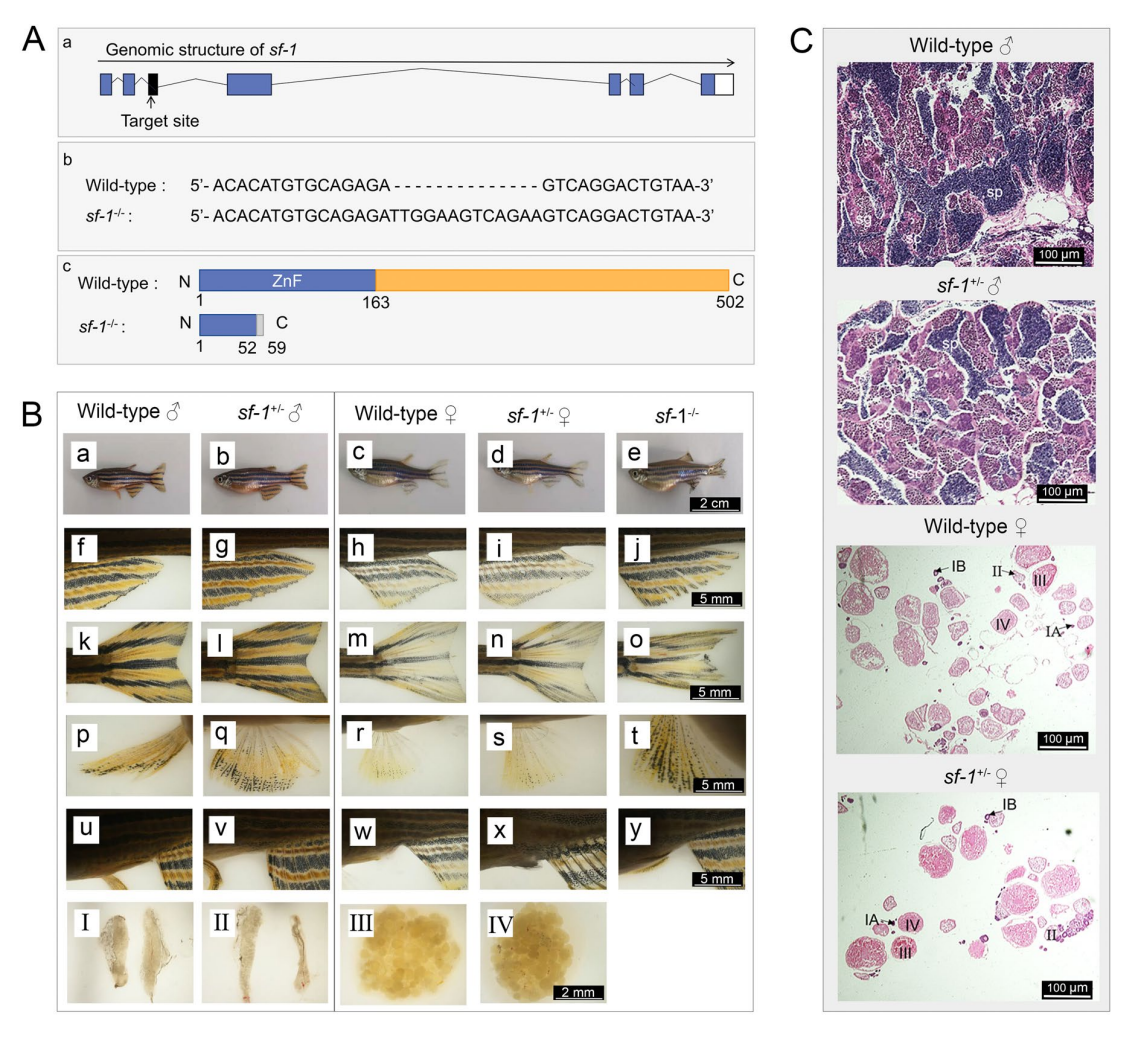
*sf-1* homozygous mutant zebrafish lost their gonads. **A** Generation of *sf-1*^*−/−*^ zebrafish via CRISPR/Cas9 technology. a. The target gene for knockout was located within exon 3. Exons are denoted by blue boxes, while introns are represented by folded lines. The white squares indicate untranslated regions. b and c. In comparison to the wild type, the mutant exhibits an insertion of 13 bases, resulting in premature termination of translation at the 59th amino acid. **B** Histological identification of the first and second sexual characteristics in wild-type and *sf-1* mutant zebrafish. The body (a–e), pelvic fin (f–j), caudal fin (k–o), anal fin (p–t), genital papilla (u–y), and gonad at the cloaca (I–IV) were observed (*n* = 10). **C** HE staining of the gonads in wild-type and *sf-1* heterozygous mutant zebrafish. sg, spermatogonia; sc, spermatocyte; sp, sperm cell. I–IV refers to oocytes of various stages

According to previous data, Zglp-1 interacted with Sf-1, which prompted us to investigate the significance of this interaction in gonad development. To accomplish our goal, we used CRISPR/Cas9 technology to delete the *sf-1* gene in zebrafish (Fig. 7A). Our findings showed a striking contrast between the gonadal phenotypes of *sf-1* heterozygous and homozygous mutants. Despite the fact that the gonads of heterozygous mutants seemed mostly unaffected, those of homozygous mutants were entirely missing (Fig. 7B, C). This striking difference underscored the critical role of Sf-1 in the development of zebrafish gonads.

Interestingly, zebrafish lacking the *zglp-1* gene retained fertile testes. This observation suggests that Zglp-1’s involvement in sexual differentiation may go beyond its interaction with Sf-1. We speculated that Zglp-1 may operate through additional, yet unidentified pathways to influence sexual differentiation. Future research should elucidate these potential alternative mechanisms and further clarify Zglp-1's multifaceted role in this complex biological process.

### Zglp-1 regulates sex differentiation by modulating proliferation and apoptosis of gonadal cells

**Fig. 8:**
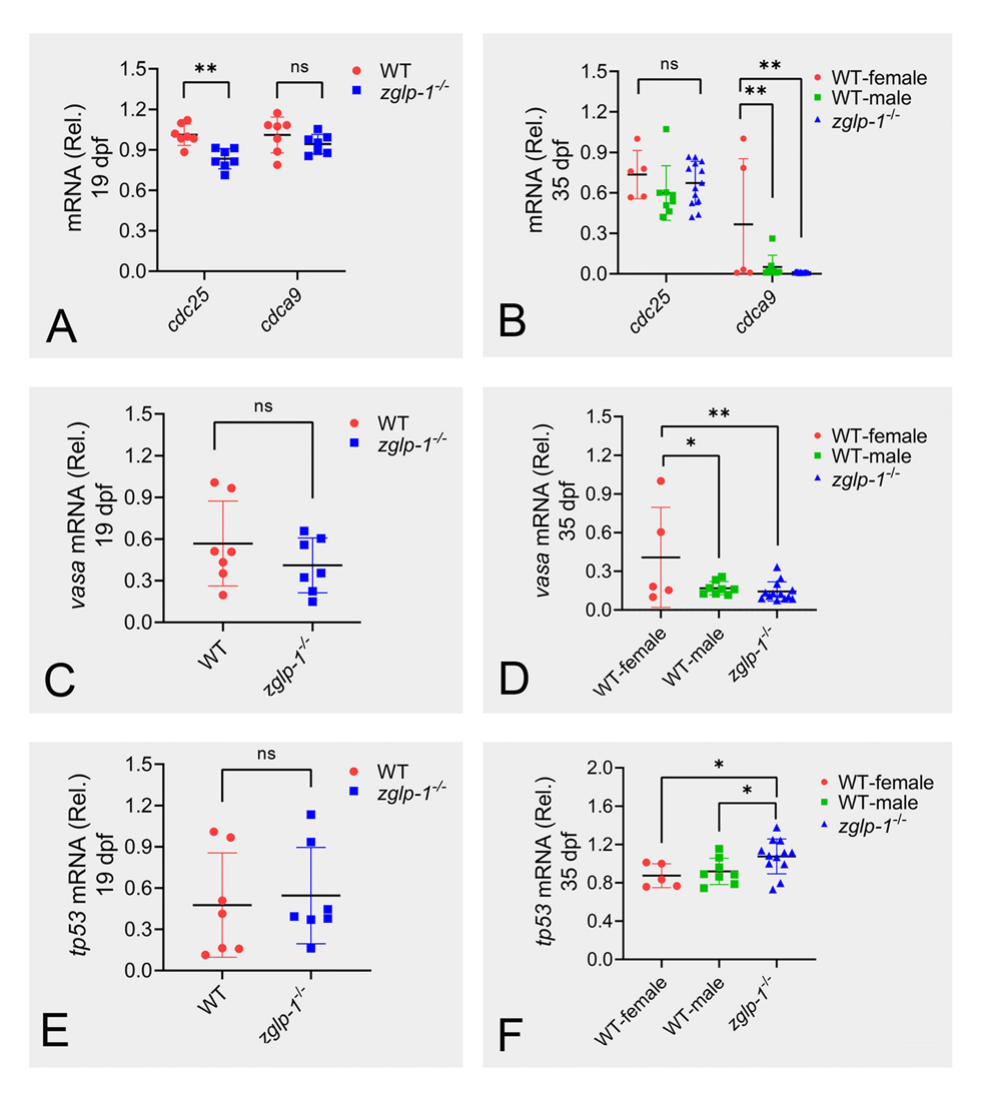
Zglp-1 regulates expression of genes involved in the proliferation and apoptosis of gonadal cells. **A**, **B** A comparative analysis was conducted to assess the expression levels of *cdc25* and *cdca9* genes in wild-type zebrafish versus *sf-1*^*−/−*^ zebrafish at 19 dpf and 35 dpf, respectively. **C**, **D** Similarly, the expression of the *vasa* gene was compared between wild-type and *sf-1*^*−/−*^ zebrafish at both 19 dpf and 35 dpf. **E**, **F** Furthermore, the expression of the *tp53* gene was evaluated in wild-type and *sf-1*^*−/−*^ zebrafish at 19 dpf and 35 dpf. *actb1* was used as a control. Data are presented as mean ± SD. The data were subjected to one-way ANOVA analysis; “ns” indicates the absence of any statistically significant difference, *P < 0.05, **P < 0.01

Chen et al. (2016) have shown that the proliferation of gonadal cells is essential for sex differentiation in zebrafish. To examine this further, we compared the expression levels of two cell division-related genes, *cdc25* and *cdca9*, in wild-type and *zglp-1* mutant zebrafish at 19 and 35 dpf. Our findings revealed that, at 19 dpf, the relative expression of *cdc25* in the *zglp-1* mutant zebrafish was notably lower than that in the wild type. Similarly, at 35 dpf, there was a significant downregulation in the expression of *cdca9* in the *zglp-1* mutant zebrafish (Fig. 8A, B).

As Tzung et al. (2015) showed that the quantity of early germ cells influenced female development in zebrafish, we aimed to investigate whether *zglp-1* deficiency had any effect on germ cell numbers. To accomplish this, we utilized real-time PCR to quantify the expression of *vasa*, a distinct marker for germ cells. Our results showed that at 35 dpf, the relative expression of the *vasa* gene in *zglp-1* homozygous mutant zebrafish was significantly reduced compared to that of wild-type female zebrafish (Fig. 8C, D).

In addition, it has been reported that germ cell apoptosis mediated by TP53 can disrupt oocyte survival by eliminating oocyte-derived signals, ultimately influencing sexual fate selection. In our study, we observed that at 35 dpf, the relative expression of *tp53* in the gonads of *zglp-1* homozygous mutant zebrafish was notably upregulated in comparison to wild-type zebrafish (Fig. 8E, F). Overall, these findings suggest that Zglp-1 may be involved in regulating sex differentiation by modulating the proliferation and apoptosis of gonadal cells.

### Zglp-1 deficiency leads to hypertrophic testis

To evaluate the impact of *zglp-1* knockout on gonad development in zebrafish, we conducted a morphological analysis of the gonads from wild-type male adults and homozygous mutant males. Our observations revealed that the testes of homozygous mutant zebrafish exhibited hypertrophy (Supplementary Fig. S3). A closer examination of the testicular section structure further highlighted notable differences. The wild-type zebrafish testes displayed intact structures (Supplementary Fig. S3B-a), while those of the *zglp-1* homozygous mutants appeared dispersed (Supplementary Fig. S3B–b). Additionally, we noticed a reduction in the percentage of sperm cells present in the testes of *zglp-1* homozygous mutants (Supplementary Fig. S3B-b).

Considering the role of the *sycp3* gene in the formation of the synaptic complex during meiosis I, we investigated its expression in zebrafish. Interestingly, our results revealed that the deletion of *zglp-1* led to a notable increase in the expression of the *sycp3* gene in the testes (Supplementary Fig. S3C). This means that in the *zglp-1* homozygous mutant, more cells in the testes failed to progress through meiosis successfully. On the whole, these findings implicated Zglp-1 in the regulation of gonad development and meiosis in zebrafish.

## Discussion

**Fig. 9:**
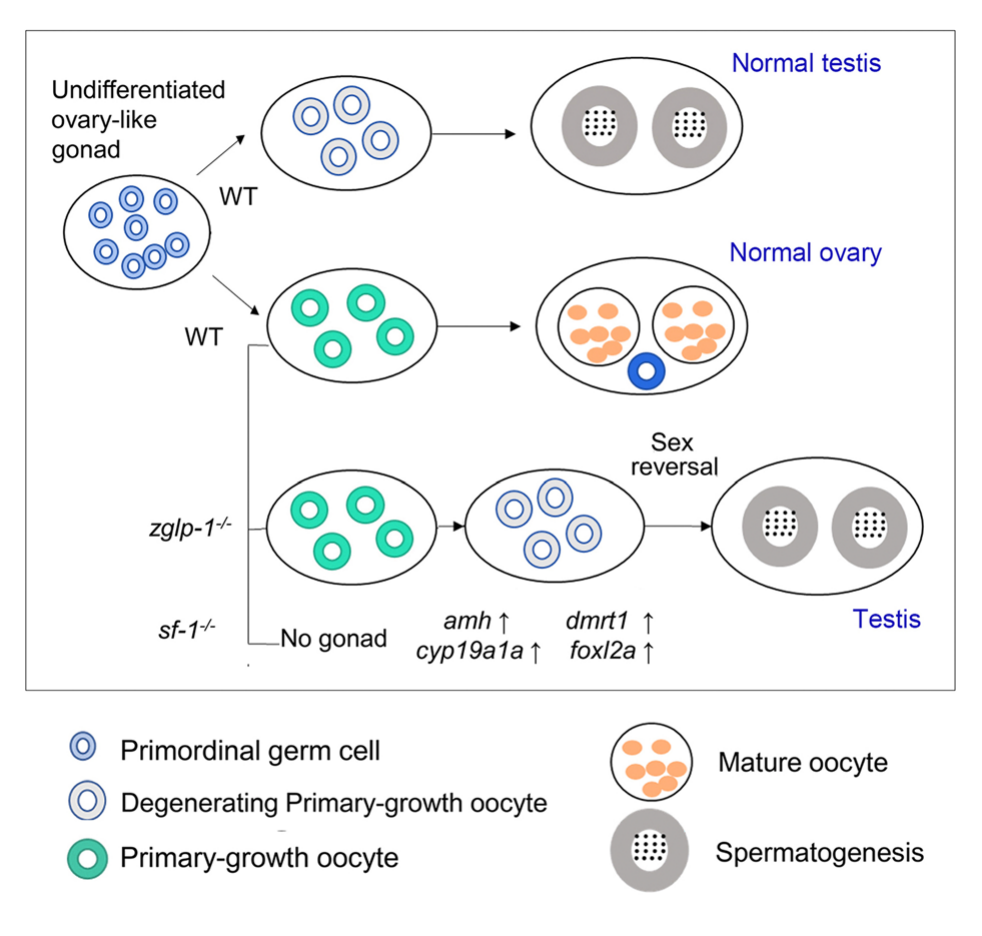
A model for the regulation of Zglp-1 in sex differentiation in zebrafish. The *zglp-1* mutant zebrafish exhibited a male-specific expression pattern, resulting in a phenotypic transition from female to male, indicating a sex reversal phenotype. *sf-1* homozygous mutant zebrafish lost their gonads

Zglp-1, which belongs to the GATA family of transcription factors, was previously recognized as a crucial factor in determining oogenic fate in mice (Li et al. 2007; Nagaoka et al. 2020; Strauss et al. 2011). Nevertheless, its specific roles in sex differentiation have remained unexplored. In our study, we developed a zebrafish model with *zglp-1* knocked out and specifically targeted the zinc finger domain for removal. Using a comprehensive analysis of these *zglp-1* mutants, we revealed that Zglp-1 deficiency significantly altered the process of sex differentiation. Remarkably, we discovered that zebrafish with the *zglp-1*^*−/−*^ mutation exclusively developed into males. This phenotypic change was not attributed to female-specific lethality but was instead caused by a female-to-male sex reversal. This reversal was marked by an increase in *amh* expression and a decrease in *cyp19a1a* expression during the early phases of gonadal development. These gene expression alterations resulted in the emergence of an entirely male zebrafish population in the *zglp-1*^*−/−*^ group. Our results, which are displayed in Fig. 9, offer groundbreaking evidence highlighting Zglp-1’s pivotal role in sex differentiation among zebrafish. This research paves the way for further exploration of the underlying mechanisms of sex determination and differentiation in vertebrates, with potential implications for comprehending reproductive disorders in humans.

*Zglp-1*, a gene identified in mice as being predominantly expressed in ovarian granulocytes (Nagaoka et al. 2020; Strauss et al. 2011), appears to have a conserved function across vertebrate species. Our study showed that the zebrafish *zglp-1* gene was highly expressed specifically in ovarian tissue. Correspondingly, Zhu et al. (2019) reported that *zglp-1* expression was predominantly found in the ovaries of the Chinese tongue sole (*Cynoglossus semilaevis*), indicating a conserved role for Zglp-1 in ovarian function across fish species. To further explore the potential involvement of *zglp-1* in gonad differentiation and development in zebrafish, we examined its expression pattern during development. Intriguingly, our results revealed that the expression pattern of *zglp-1* resembled that of *amh*, a gene recognized for its pivotal role in male differentiation. In particular, AMH, which is a component of the transforming growth factor beta (TGF-beta) superfamily, is expressed in Sertoli cells and is responsible for the regression of Müllerian ducts in mammals (De et al. 1998; Dimitriadis et al. 2015; Josso et al. 1998; Rouiller-Fabre et al. 1998; Saitou et al. 2009; Wang et al. 2016). Recent knockout studies in zebrafish confirmed the necessity of *amh* for male differentiation (Lin et al. 2017; Webster et al. 2017). Our finding that *zglp-1* and *amh* genes exhibited similar expression patterns during zebrafish development implies that *zglp-1* may also be involved in the process of gonad differentiation and development. While the specific mechanisms whereby Zglp-1 regulates gonad development remain to be elucidated, our results provide novel insights into the conserved roles of Zglp-1 in ovarian function and highlight its potential involvement in sex differentiation across vertebrates.

The observation that *zglp-1*^*−/−*^ zebrafish undergo female-to-male sex reversal, resulting in fertile males, while *Zglp-1* knockout homozygous mice do not exhibit sex reversal and are infertile, reveals significant differences in the function of Zglp-1 between these two species (Rouiller-Fabre et al. 2017). These differences suggest that the role of Zglp-1 in sex differentiation may have undergone changes during evolution. Such evolutionary divergence makes zebrafish an attractive model for studying the molecular regulation of sex differentiation, as zebrafish exhibit a more plastic sex determination system than mammals. A unique feature of zebrafish sex determination is the absence of sex chromosomes (Traut et al. 2001; Wallace et al. 2003). By contrast, sex differentiation in zebrafish is influenced by a multitude of genetic and environmental factors. It has been shown that external cues such as light, temperature, and food affect sex differentiation in zebrafish (Hsu et al. 2018; Ribas et al. 2017). However, genetic factors remain the fundamental basis for sex determination and differentiation. There have been numerous reports of genetic mutations leading to sex reversal in zebrafish. For example, homozygous mutations in *fancl* or *cyp19a1a* resulted in female-to-male sex reversal (Rodríguez-Marí et al. 2010; Wu et al. 2020). In a similar fashion, *amh*^*−/−*^ zebrafish were prone to develop as females (Lin et al. 2017). In this study, the discovery of female-to-male sex reversal in *zglp-1* homozygous mutant zebrafish further broadens our understanding of the genetic factors involved in sex differentiation. Overall, these findings underscore the intricate nature and adaptability of sex determination in zebrafish, emphasizing the crucial role of genetic components in shaping this process.

The results of our study suggest a complex interaction between Zglp-1, Sf-1, and sex differentiation markers in zebrafish. Compared with wild-type females, zebrafish lacking zglp-1 (*zglp-1*^*−/−*^) at 35 dpf exhibited elevated expression of the male-specific marker genes *amh* and *dmrt1* while displaying reduced expression of the female-specific marker genes *cyp19a1a* and *foxl2a*. This suggests a tendency toward male gonadal development in the absence of Zglp-1. SF-1, belonging to the nuclear receptor subfamily 5 group A member 1, plays a pivotal role in steroidogenesis and reproduction in mammals, as reported in various studies (Anamthathmakula et al. 2019; Jeyasuria et al. 2004; Lavorgna et al. 1991; Ozisik et al. 2003; Suntharalingham et al. 2015). Nevertheless, its function appears to differ in zebrafish and other fish species. Although *Sf-1* homozygous mutant mice exhibited gonadal hypoplasia (Meinsohn et al. 2019; Młynarczuk et al. 2010), disruption of *Sf-1* in Nile tilapia led to female-to-male sex reversal (Fang et al. 2018; Xie et al. 2016; Zhang et al. 2017). This indicates that Sf-1 may have distinct roles in different vertebrates. Interestingly, Sf-1 was found to activate the *amh* promoter in zebrafish (Schteingart et al. 2019), but Zglp-1 could not. Furthermore, in the present study, Zglp-1 was shown to inhibit the transcriptional activation of *amh* by Sf-1. Hence, Zglp-1 may play a role in promoting female sex differentiation by antagonizing the action of Sf-1 on *amh* expression. The direct interaction between Zglp-1 and Sf-1, particularly through their zinc finger domains, provides a mechanism for this inhibition. However, the complete disappearance of gonads in *sf-1* knockout zebrafish indicated that Sf-1 is essential for gonad development, but its role in sex differentiation may be more complex. The incomplete sex reversal observed in *amh* gene knockout zebrafish further indicates that additional factors are involved in sex determination and differentiation (Lin et al. 2017). It appears that Zglp-1 plays a regulatory role in sex differentiation in zebrafish, presumably by interacting with Sf-1 and modulating its activity on *amh* expression. Nevertheless, the exact mechanism through which Zglp-1 governs sex differentiation has not yet been fully elucidated, and further investigations are warranted to illuminate the functions of Zglp-1, Sf-1, and other contributing factors in this intricate process.

Previous studies have shown that decreased proliferation of gonad cells in *nr0b1* mutant zebrafish can lead to sex reversal, pushing the gonads toward male development (Chen et al. 2016). Our findings revealed a significant downregulation of *cdca9* expression in *zglp-1* homozygous mutant zebrafish at 35 dpf. This suggests that the *zglp-1* gene mutation may impair the proliferative capacity of gonadal cells, potentially contributing to the sex reversal observed in *zglp-1* mutants. Similarly, an *Igf3* gene mutation in zebrafish has been shown to decrease germ cell proliferation and result in female-to-male sex reversal (Wood et al. 2005). Vasa, a key player in the migration, survival, and maintenance of germ cells, serves as a germ cell marker gene. Our study found that the expression of *vasa* was significantly downregulated in *zglp-1* homozygous mutant zebrafish compared to wild-type females. This suggests that the *zglp-1* mutation may reduce the germ cell population, providing another potential explanation for the sex reversal phenotype in *zglp-1* mutants. Nevertheless, recent studies on zebrafish have revealed that a mere 35% reduction in germ cells does not always lead to sex reversal (Dai et al. 2015). Apoptosis of germ cells has been identified as a crucial factor in male zebrafish development (Tzung et al. 2015). For example, an elevation in germ cell apoptosis and oocyte failure was documented in *fancl*^*−/−*^ zebrafish, ultimately leading to female-to-male sex reversal (Rodríguez-Marí et al. 2010; Ye et al. 2020). *Tp53*, a gene recognized for its role in inhibiting cell division and triggering apoptosis, can be used as a marker for apoptosis. In our investigation, we observed notable upregulation of *tp53* expression in the gonads of *zglp-1*^*−/−*^ zebrafish at 35 dpf. Therefore, Zglp-1 may influence sex differentiation by regulating apoptosis in gonad cells. It should be noted that our conclusions are primarily based on real-time PCR data, and the introduction of *tp53* mutants into future studies would further strengthen our findings. Additionally, studies have indicated that the occurrence of female-to-male sex reversal in zebrafish *fancl* mutants is linked to hindered meiotic progression in oocytes (Rodríguez-Marí et al. 2010). Our transcriptome analysis revealed numerous differentially expressed genes linked to meiosis. Moreover, ovaries from *zglp-1* heterozygous mutants exhibited an increased proportion of oocytes, suggesting a potential arrest at a specific meiotic stage.

In summary, our study explored the significant function of Zglp-1 in zebrafish sex differentiation. Our results implied that Zglp-1, as a zinc finger protein, interacted with Sf-1, impeding its capacity to stimulate the expression of *amh*, a pivotal gene in the sex differentiation process. This interaction eventually resulted in a male-like expression pattern in *zglp-1*^*−/−*^ zebrafish, leading to female-to-male sex reversal. Furthermore, the study indicated that Zglp-1 may affect cell proliferation and apoptosis of gonadal cells, which could be a mechanism for determining sex fate. Nevertheless, further investigations are needed to fully understand the mechanism by which Zglp-1 regulates sex differentiation. This includes identifying other proteins that bind to Zglp-1 in addition to Sf-1 and determining the target gene for Zglp-1 as a transcription factor. Such investigations would lay the groundwork for a deeper and more refined understanding of sex differentiation mechanisms in zebrafish, and possibly in other vertebrate species.

## Supplementary Information

Below is the link to the electronic supplementary material.

Supplementary file1 (DOCX 1143 KB)

## Data Availability

All relevant data are available from the authors upon request and the corresponding author will be responsible for replying to the request.
