# Supplementary material for: Zglp-1 is a novel essential transcriptional regulator for sex reversal in zebrafish
Source: Mar Life Sci Technol. 2025 May 12;7(2):256–70. doi: 10.1007/s42995-025-00299-5 (PMC12102022; doi:10.1007/s42995-025-00299-5)
Supplement: Supplementary file 1 — Supplementary file1 (DOCX 1143 KB) [file 42995_2025_299_MOESM1_ESM.docx]

**Supplementary Table**

**Table S1. Sequences of the primers used in this study**

| Name and brief Description | Primer sequence (5’-3’) |
| --- | --- |
| **CRISPR/Cas9**  P1(*zglp-1* F)  P2(*zglp-1* R)  P3(*sf-1* F)  P4(*sf-1* R)  **Real-time PCR** | TCCATCCACAGGGAAATCTTGTGACATCACTTCCTGTCT  GAACCTCCTCAGTGGAGTATGAATGCGTCTACACCACCT  AAACACAACGGCTTATGGAAATACA  AGTATGCAACATAAATAAACAACTAGACA |
| P5（*actin* F） | CGAGCAGGAGATGGGAACC |
| P6（*actin* R） | CAACGGAAACGCTCATTGC |
| P7(*zglp-1* F) | CCGACTGCTCATCACCTCCAAC |
| P8(*zglp-1* R) | GGTGCGGCAAGATGCACAGAT |
| P9(*cyp19a1a* F) | CCTGTTGTCTCCTACTGTCGGTTCA |
| P10(*cyp19a1a* R) | GACCCAGTTTACTTCCAAAGCGTGA |
| P11(*amh* F) | CTGTGATGCTCCTGTTCAGTGTCAA |
| P12(*amh* R) | AAGGTGAATGTGGCCATGACTCCAT |
| P13（*dmrt1* F） | ATGGCAGAGCAGAACGATTTACTG |
| P14（*dmrt1* R） | TAGTCCCACAACAGCATGGAGAT |
| P15（*foxl2a* F） | CTACGAGAAGAACAAGAAGGGATGG |
| P16（*foxl2a* R） | CTGACAGGAGGTATATGACATGGG |
| P17（*cdc25* F） | TCTCCCCACCACCCACCAAT |
| P18（*cdc25* R） | CCCTCCAGAAAGCCATCATCGT |
| P19（*cdca9* F） | TGTTGGCGAGGTGACGATGG |
| P20（*cdca9* R） | TTTTGGCATTGATGGTTGAGGC |
| P21（*vasa* F） | TAGGAAGAACTGGACGCTGTGGG |
| P22（*vasa* R） | GCTCGGGGCAGATGTTTGTGAT |
| P23（*tp53* F） | CGCAGGCCCATCCTCACAAT |
| P24（*tp53* R） | TTGCTCCCCTCAGGTCGTAATGTA |
| P25(*sycp3* F) | ATGGCCGCTGCAGGCAGAAA |
| P26(*sycp3* R) | TCAGAACAGCATGGACTGAAGAG |
| ***In Situ* Hybridization**  P27 (*zglp-1* F)  P28 (*zglp-1* R) | ATGCTGGGCTGGATTGTCC  CTACCAGCCGCCGCTCTT |
| **Luciferase assay** |  |
| P29(*zglp-1-ZnF* F) | tagtccagtgtggtggaattcCAGCCCCGTCCTGCACGC |
| P30(*zglp-1-ZnF* R) | aacgggccctctagactcgagCTACCAGCCGCCGCTCTT |
| P31(*zglp-1-N* F) | tagtccagtgtggtggaattcATGCTGGGCTGGATTGTCC |
| P32(*zglp-1-N* R) | aacgggccctctagactcgagCTTCCTCCGGCGGCACAC |
| **Subcellular co-location** |  |
| P33(*Zglp-1-Egfp* F) | tagtccagtgtggtggaattcATGCTGGGCTGGATTGTCC |
| P34(*Zglp-1-eGFP* R) | aacgggccctctagactcgagCTACCAGCCGCCGCTCTT |
| P35(*Sf-1-mCherry* F) | tagtccagtgtggtggaattcATGGACTACAGTTATGATGCGGAC |
| P36(*Sf-1-mCherry* R) | aacgggccctctagactcgagTCAGATGCAGGTTCTCTTGGC |
| **Co-IP** |  |
| P37(*pCMV-HA-zglp-1* F) | tagtccagtgtggtggaattcATGCTGGGCTGGATTGTCC |
| P38(*pCMV-HA- zglp-1* R) | aacgggccctctagactcgagCTACCAGCCGCCGCTCTT |
| P39(*pCMV-Myc-sf-1* F) | tagtccagtgtggtggaattcATGGACTACAGTTATGATGCGGAC |
| P40(*pCMV-Myc-sf-1* R) | aacgggccctctagactcgagTCAGATGCAGGTTCTCTTGGC |
| P41(*pCMV-HA- zglp-1-ZnF* F) | tccaagcttctgcaggaattcCAGCCCCGTCCTGCACGCAGC |
| P42(*pCMV-HA- zglp-1-ZnF* R) | accgggcccactagttctagaCTACCAGCCGCCGCTCTTCAT |
| P43(*pCMV-HA- zglp-1-N* F) | tccaagcttctgcaggaattcATGCTGGGCTGGATTGTCCGT |
| P44(*pCMV-HA- zglp-1-N* R) | accgggcccactagttctagaCTTCCTCCGGCGGCACACAC |
| P45(*pCMV-Myc-sf-1-ZnF* F) | tccaagcttctgcaggaattc ATGGACTACAGTTATGATGCGGACC |
| P46(*pCMV-Myc-sf-1-ZnF* R) | accgggcccactagttctagaGCGTTCGTAATCGGTGGGTGCCACA |
| P47(*pCMV-Myc-sf-1-C* F) | tccaagcttctgcaggaattcAGCCTCTACGCATCCAGCTCCCT |
| P48(*pCMV-Myc-sf-1-C* R) | accgggcccactagttctagaTCAGATGCAGGTTCTCTTGGCATGC |

**Supplementary Figures**

**
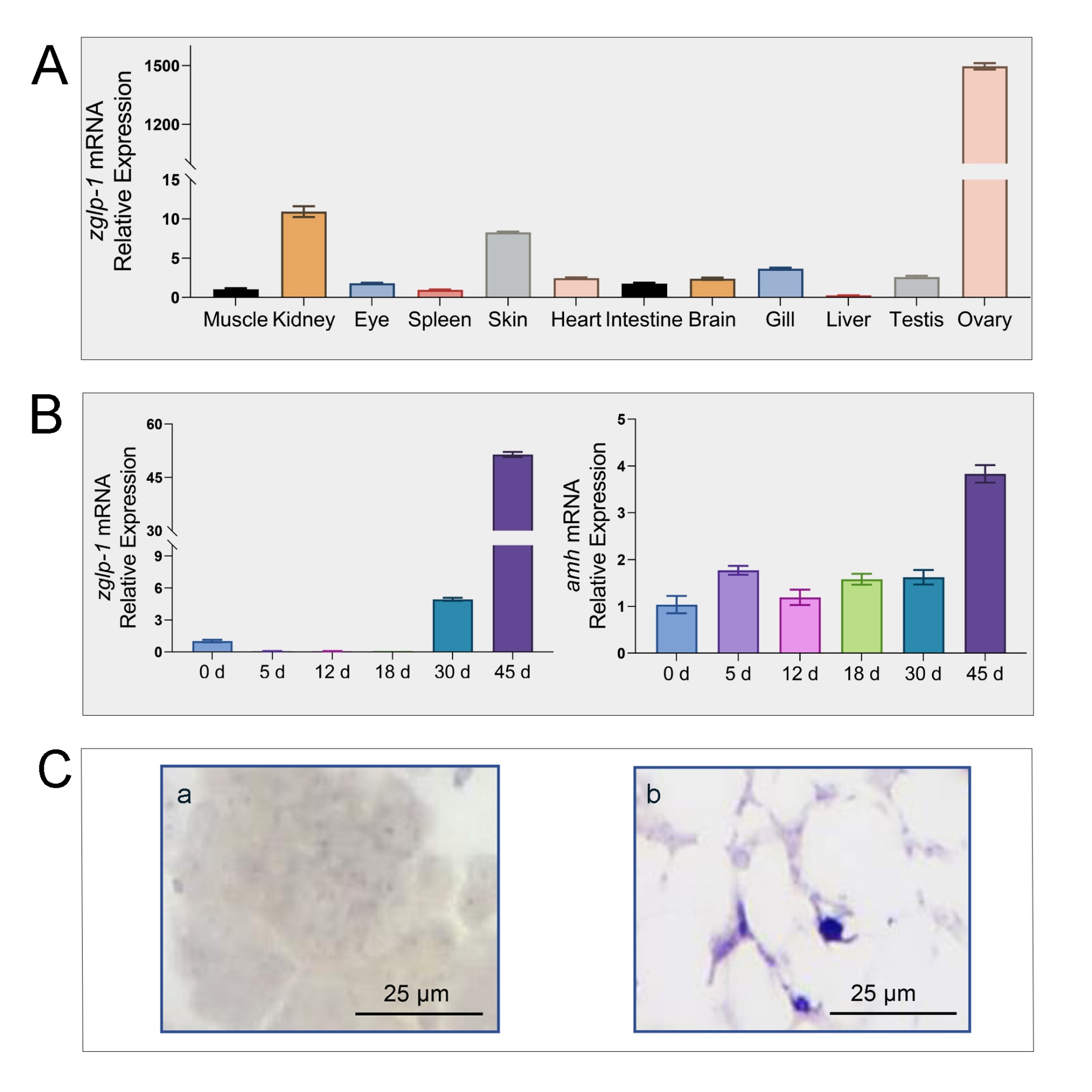
**

**Fig. S1 zglp-1 is highly expressed in the zebrafish ovary.** (**A**) The expression of *zglp-1* in different tissues in zebrafish detected by quantitative RT-PCR technology. *zglp-1* was highly expressed in the adult ovary. (**B**) Expression of zebrafish z*glp-1* and *amh* genes in different developmental stages. (**C**) Section ISH of zebrafish z*glp-1*. a: testis; b: ovary. The *β-actin* was used as loading control. Data are presented as mean ± SD.­­­­­­


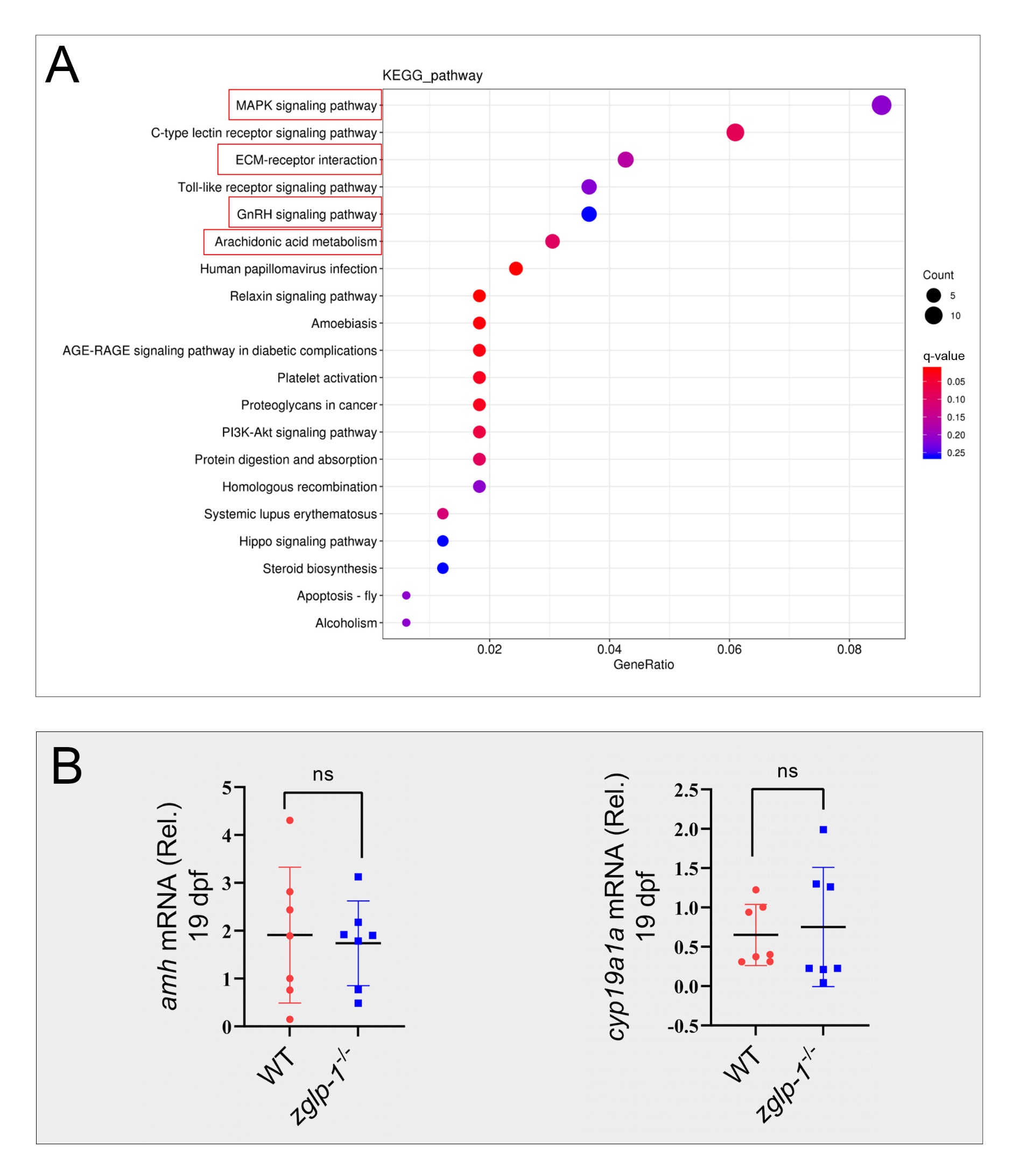


**Fig. S2 Comparison of the expression of genes in wild type zebrafish and zglp-1 homozygous mutant zebrafish at 19 dpf.** (**A**) KEGG analysis of differentially expressed genes in wild type zebrafish and *zglp-1*^-/- +2^ zebrafish at 19 dpf. The size and color of dots show the number of differentially expressed genes and q-value, respectively. The path represented by the graph near the upper right corner is of significant reference value. (**B**) The expression of *amh* and *cyp19a1a* of wild type and *zglp-1* homozygous mutant zebrafish at 19 dpf detected by Real-time PCR. *β-actin* was used as the loading control. Data are presented as mean ± SD. Data were analyzed by One-way ANOVA (ns not significant).


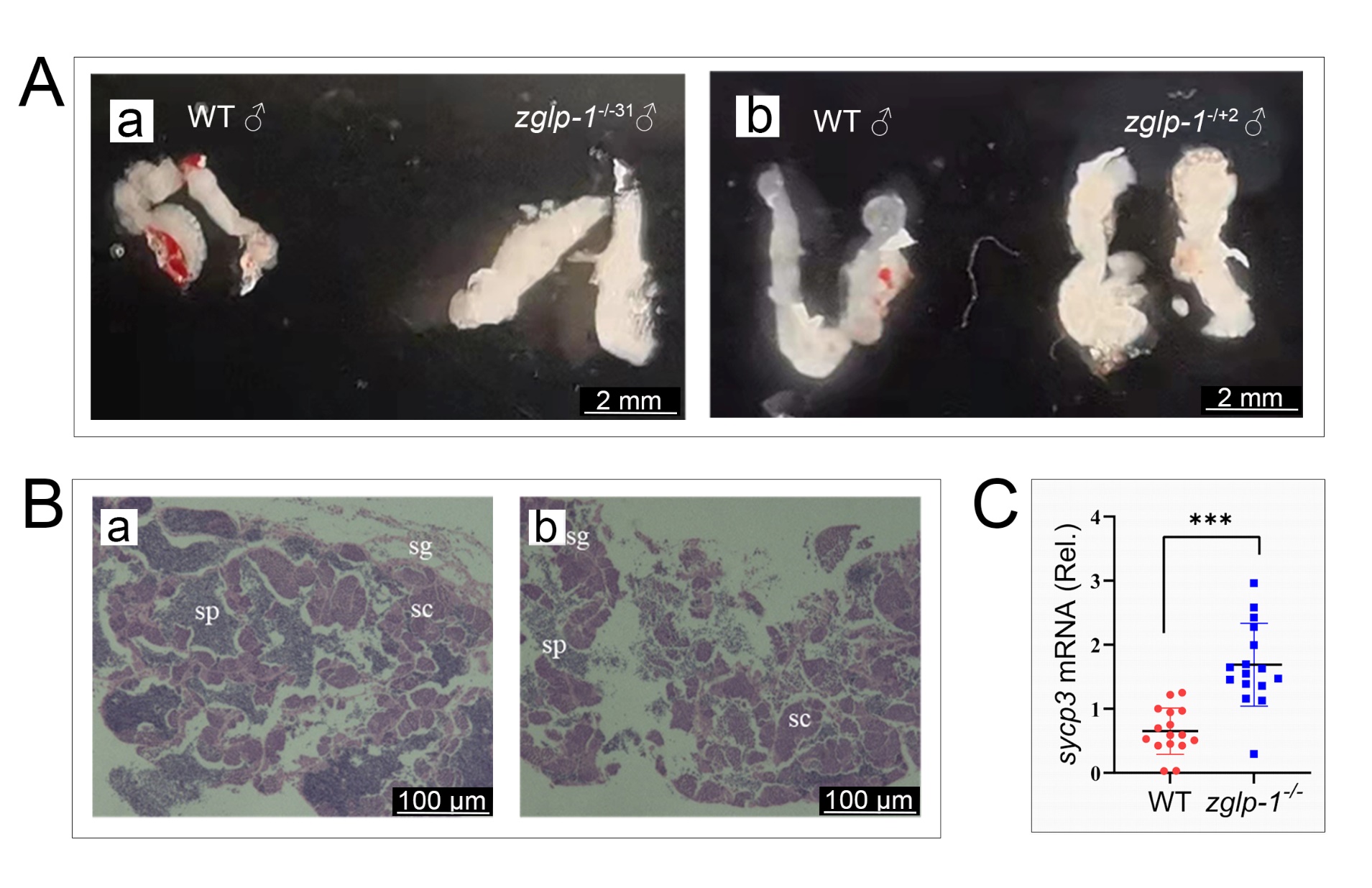


**Fig. S3 Zglp-1 deficiency leads to a hypertrophy testis.** (**A**) Comparison of testis between wild type and *zglp-1* homozygous mutant male zebrafish. (**B**) HE staining of testis section of wild type (a) and *zglp-1* homozygous mutants (b). sg: spermatogonia, sc: spermatocytes, sp: sperm. (**C**) The *sycp3* gene was detected in testis of wild type and *zglp-1* homozygous mutants by Real-time PCR. *β-actin* was used as the control. Data are presented as mean ± SD. Data were analyzed by One-way ANOVA. *** *P* <0.001.
